# Involvement of a periodontal pathogen, *Porphyromonas gingivalis *on the pathogenesis of non-alcoholic fatty liver disease

**DOI:** 10.1186/1471-230X-12-16

**Published:** 2012-02-16

**Authors:** Masato Yoneda, Shuhei Naka, Kazuhiko Nakano, Koichiro Wada, Hiroki Endo, Hironori Mawatari, Kento Imajo, Ryota Nomura, Kazuya Hokamura, Masafumi Ono, Shogo Murata, Iwai Tohnai, Yoshio Sumida, Toshihide Shima, Masae Kuboniwa, Kazuo Umemura, Yoshinori Kamisaki, Atsuo Amano, Takeshi Okanoue, Takashi Ooshima, Atsushi Nakajima

**Affiliations:** 1 Department of Gastroenterology, Yokohama City University Graduate School of Medicine, Yokohama, Japan; 2 Department of Pediatric Dentistry, Graduate School of Dentistry, Osaka University, Suita, Osaka, Japan; 3 Department of Pharmacology, Graduate School of Dentistry, Osaka University, Suita, Osaka, Japan; 4 Department of Pharmacology, Hamamatsu University School of Medicine, Hamamatsu, Japan; 5 Department of Gastroenterology and Hepatology, Kochi Medical School, Kochi, Japan; 6 Department of Oral and Maxillofacial Surgery, Yokohama City University Graduate School of Medicine, Yokohama, Japan; 7 Center for Digestive and Liver Diseases, Nara City Hospital, Nara, Japan; 8 Hepatology Center, Saiseikai Suita Hospital, Suita, Japan; 9 Department of Preventive Dentistry, Graduate School of Dentistry, Osaka University, Suita, Osaka, Japan; 10 Department of Pharmacology, Graduate School of Dentistry, Osaka University, 1-8 Yamada-oka, Suita, Osaka 565-0871, Japan

**Keywords:** Non-alcoholic fatty liver disease (NAFLD), Non-alcoholic steatohepatitis (NASH), *P. gingivalis*, Oral bacteria, Insulin resistance

## Abstract

**Background:**

Non-alcoholic fatty liver disease (NAFLD) is a hepatic manifestation of metabolic syndrome that is closely associated with multiple factors such as obesity, hyperlipidemia and type 2 diabetes mellitus. However, other risk factors for the development of NAFLD are unclear. With the association between periodontal disease and the development of systemic diseases receiving increasing attention recently, we conducted this study to investigate the relationship between NAFLD and infection with *Porphyromonas gingivalis *(*P. gingivalis*), a major causative agent of periodontitis.

**Methods:**

The detection frequencies of periodontal bacteria in oral samples collected from 150 biopsy-proven NAFLD patients (102 with non-alcoholic steatohepatitis (NASH) and 48 with non-alcoholic fatty liver (NAFL) patients) and 60 non-NAFLD control subjects were determined. Detection of *P. gingivalis *and other periodontopathic bacteria were detected by PCR assay. In addition, effect of *P. gingivalis*-infection on mouse NAFLD model was investigated. To clarify the exact contribution of *P. gingivalis*-induced periodontitis, non-surgical periodontal treatments were also undertaken for 3 months in 10 NAFLD patients with periodontitis.

**Results:**

The detection frequency of *P. gingivalis *in NAFLD patients was significantly higher than that in the non-NAFLD control subjects (46.7% vs. 21.7%, odds ratio: 3.16). In addition, the detection frequency of *P. gingivalis *in NASH patients was markedly higher than that in the non-NAFLD subjects (52.0%, odds ratio: 3.91). Most of the *P. gingivalis *fimbria detected in the NAFLD patients was of invasive genotypes, especially type II (50.0%). Infection of type II *P. gingivalis *on NAFLD model of mice accelerated the NAFLD progression. The non-surgical periodontal treatments on NAFLD patients carried out for 3 months ameliorated the liver function parameters, such as the serum levels of AST and ALT.

**Conclusions:**

Infection with high-virulence *P. gingivalis *might be an additional risk factor for the development/progression of NAFLD/NASH.

## Background

Almost one-quarter of the adults in the world population show excessive hepatic fat accumulation, and non-alcoholic fatty liver disease (NAFLD) is the most common form of chronic liver disease encountered in many industrialized countries [1,2]. NAFLD represents a wide spectrum of conditions ranging from non-alcoholic fatty liver (NAFL) to non-alcoholic steatohepatitis (NASH) [1-3]. The former generally shows a non-progressive clinical course, while the latter is a more serious form of NAFLD and may progress to cirrhosis or end-stage liver disease [1-6]. Many risk factors for the development of NAFLD have been proposed, such as obesity, diabetes, insulin resistance, oxidative stress and inflammation [1-6]. However, it is still unclear whether other risk factors may also be involved in the pathogenesis and progression of NAFLD. In addition, the factors involved in the progression of NAFL to NASH are also not fully understood. Therefore, identification of factors responsible for the progression of NASH would be helpful in the prevention and designing of strategies for the treatment of NASH.

Chronic marginal periodontitis occurs worldwide and is among the most prevalent of microbial diseases in humans [7]. Periodontal inflammation often leads to superficial ulcers on the gingival sulcus, where blood capillaries are exposed to microbial biofilms [8]. It is well known that periodontal pathogens are translocated and released from the sulcus into the bloodstream, and such transient bacteremia has been demonstrated in clinical trials to occur after preventive dental procedures and periodontal therapy, including tooth brushing, chewing, subgingival irrigation, periodontal treatment, and dental extractions, at reported frequencies ranging from 17% to 100% in infected individuals [9-11]. *Porphyromonas gingivalis *(*P. gingivalis*) is well known as a major causative agent of periodontitis [12-14]. Recent reports suggest that infection with *P. gingivalis *is associated with several systemic diseases, including cardiovascular diseases, preterm low birth weight, rheumatoid arthritis, and diabetes mellitus (DM) [12-14]. However, there are no reports until date concerning the involvement *P. gingivalis *infection in the pathogenesis and progression of NAFLD. Therefore, we conducted this retrospective observational study to investigate the relationship between NAFLD and infection with *P. gingivalis*.

## Methods

### Patients and control subjects

This study was conducted with the approval of the Ethics Committees of Yokohama City University, Osaka University, and Nara City Hospital. A total of 150 Japanese NAFLD patients (102 with NASH and 48 with NAFL) and 60 control subjects (non-NAFLD) from similar socioeconomic strata were recruited. After provision of adequate explanation, written informed consent for participation in this study was obtained from all the subjects prior to the sample collections. All control subjects (non-NAFLD) were confirmed to have normal liver function (aspartate aminotransferase (AST), alanine aminotransaminase (ALT) levels within normal range), no evidence of viral hepatitis and no evidence of the presence of criteria of the metabolic syndrome, such as diabetes mellitus and high serum cholesterol (see Table 1). They were also confirmed to not have a history of habitual alcohol drinking-in this context, all subjects with a history of excessive alcohol consumption (> 20 g/day) were excluded. Other exclusion criteria for NAFLD patients were a history of use of drugs associated with fatty liver diseases and a history of other hepatic diseases, such as chronic hepatitis C, hepatitis B (serum positive for hepatitis B surface antigen), autoimmune hepatitis, primary biliary cirrhosis (PBC), sclerosing cholangitis, hemochromatosis, alpha-antitrypsin deficiency, Wilson's disease, hepatic injury caused by substance abuse [15-18]. None of the patients had any clinical evidence of hepatic decompensation, e.g. hepatic encephalopathy, ascites, variceal bleeding, or elevation of the serum bilirubin level to more than twofold the upper limit of normal [15-17].

**Table 1:** Summary of the data of the NAFLD and healthy subjects

	Healthy (n = 60)	NAFLD (n = 150)	*P *value
Age (mean ± SE)	52.9 ± 2.4	54.6 ± 1.2	0.4609
Gender (M : F)	29 : 31	64 : 86	0.5387
AST (mean ± SE)	20.7 ± 0.6	49.0 ± 2.3	< 0.0001
ALT (mean ± SE)	18.3 ± 0.9	70.1 ± 3.6	< 0.0001
DM (Y : N)	0 : 60	35 : 105	< 0.0001
Histopathological Findings			
Steatosis			
1	NA	53	
2	NA	57	
3	NA	40	
Inflammation			
0	NA	28	
1	NA	97	
2	NA	22	
3	NA	3	
Ballooning			
0	NA	50	
1	NA	52	
2	NA	48	
Fibrosis			
0	NA	49	
1	NA	54	
2	NA	24	
3	NA	19	
4	NA	4	

DM: with (Y) or without (N) diabetes mellitus. NA: not applicable for biopsy.

### Pathology

Liver specimens were obtained from NAFLD patients for the purpose of diagnosis and staging for NASH, with an 18-gauge needle biopsy apparatus (Pro-Mag, Medical Device Technologies, Gainsville, FL, USA). In our present study, 102 were NASH and 48 were NAFL (non-alcoholic fatty liver). On NASH patients, their ALT/AST levels were much increased and other many abnormal symptoms were observed. So, all of NASH patients were applied the biopsy. Among the 48 NAFL patients, 36 cases showed the abnormal ALT/AST levels, and other abnormal symptoms were observed. Therefore, they were also applied the biopsy. Other 12 NAFL patients, their ALT levels were almost normal range. It was reported that the patients of NAFLD with normal ALT level may also have histological features at risk for disease progression [19-21]. Therefore, we performed liver biopsy for the purpose of diagnosing and staging for NASH, although the ALT level was in normal range. However, the case of application for the biopsy was strictly defined by the criteria follows; The NAFLD patients were suspected that 1) the existence of severe steatosis (such as persist evidence of severe steatosis at ultrasonography and/or CT) or 2) the fibrosis was progressed (such as platelet count was low and/or serum albmin was low and/or the hyaluronic acid was high and/or the type IV collagen 7s domain was high and/or the liver stiffness was high by transient elastography). In fact, the 12 NAFL patients were fit the cases described above. We excluded unclear borderline cases in this study, because they were not performed the biopsy. Therefore, total 150 NADLF patients (102 NASH + 36 abnormal ALT/AST-NAFL + 12 normal ALT/AST-NAFL) were applied the biopsy.

We did not perform any biopsies on control subjects, because their ALT/AST levels are normal and they have no abnormal symptoms.

The biopsy specimens were obtained from the same lobe to enable obtainment of a sufficient sample size for analysis, and stained with hematoxylin-eosin, reticulin, and Masson trichrome stains. The histopathological findings were evaluated and confirmed as NAFLD/NASH according to the criteria of Matteoni et al. [15]. The criterion for the diagnosis of NAFLD was; macrovesicular steatosis affecting at least 5% of the hepatocytes, with the cases being further classified as steatosis or steatohepatitis. In addition to the presence of steatosis, the minimum criteria for the diagnosis of steatohepatitis included the presence of lobular inflammation and either ballooning of cells or perisinusoidal/pericellular fibrosis in zone 3 of the hepatic acini [15]. The degree of steatosis was assessed as follows, based on the percentage of hepatocytes containing macrovesicular fat droplets: grade 0, no steatosis; grade 1, < 33% hepatocytes containing macrovesicular fat droplets; grade 2, 33%-66% hepatocytes containing macrovesicular fat droplets; grade 3, > 66% hepatocytes containing macrovesicular fat droplets. The severity of the necroinflammatory activity was assessed as follows: grade 1, occasional ballooned hepatocytes (mainly zone 3), scattered intraacinar neutrophils ± lymphocytes, no or mild portal inflammation; grade 2, obvious ballooning degeneration in zone 3, intra-acinar neutrophils, mild to moderate portal and intra-acinar inflammation; grade 3, widespread ballooning, intra-acinar inflammation, accumulation of neurtophils near the ballooned hepatocytes, mild to moderate portal inflammation [15-18].

### Clinical and laboratory evaluation

Blood samples were obtained from the patients after overnight fasting (12 h) for measurement of the serum levels of AST, ALT, gamma glutamyltranspeptidase (GGT), choline esterase, albumin, triglyceride, high density lipoprotein (HDL) cholesterol, low density lipoprotein (LDL) cholesterol, iron, ferritin, insulin, type IV collagen 7s and hyaluronic acid (HA) concentration, fasting blood glucose, and hematological parameters, including the red blood cell count, hematocrit, and hemoglobin. Serum insulin levels were measured by radioimmunoassay.

Other laboratory biochemical parameters were measured with a conventional automated analyzer. IR was calculated by the modified homeostasis model assessment of insulin resistance (HOMA-IR) using the formula: HOMA-IR = fasting insulin (lU/ml) × plasma glucose (mg/dl)/405 [22].

### Detection of P. gingivalis and other periodontopathic bacteria from saliva samples

The specimens were processed for PCR assay using a previously reported method [23]. Briefly, bacterial cells were collected in micro-centrifuge tubes after centrifugation of 500 μl of saliva at 16,000 × *g *for 5 min. Next, 80 μl of a nuclear lysis buffer (Promega, Madison, WI, USA) was added, and the mixture was incubated at 80°C for 5 min, followed by the addition of 60 μl of protein precipitation solution (Promega). The proteins were removed by centrifugation at 16,000 × *g *for 3 min, and the DNA was purified by phenol-chloroform extraction and ethanol precipitation. The extracted DNA was then dissolved in 50 μl of TE buffer (10 mM Tris-HCl, 1 mM EDTA [pH 8.0]).

First, we confirmed the appropriate extraction of DNA by PCR using a ubiquitous primer set (5'-AGA GTT TGA TCC TGG CTC AG-3' and 5'-GGC TAC CTT GTT ACG ACT T-3') [23]. Then, *P. gingivalis *was detected using a PCR method with a *P. gingivalis*-specific primer set (5'-TGT AGA TGA CTG ATG GTG AAA ACC-3' and 5'-ACG TCA TCC CCA CCT TCC TC-3'), as described previously [24]. *P. gingivalis*-positive specimens were further analyzed to differentiate among their *fimA *genotypes using a PCR method with specific primer sets for each *fimA *type as previously reported [23,25,26]. Genomic DNA samples extracted from *P. gingivalis *strains ATCC33277 (type I), OMZ314 (II), 6/26 (III), HG564 (IV), HNA99 (V), and HG1691 (Ib) were used as positive controls.

Detection of other periodontal bacteria, such as *Treponema denticola, Prevotella intermedia, Tannerella forsythia, Aggregatibactor actinomycetemcomitans, Campyrobacter rectus*, was also performed according to a previously described method [27].

### Animal experiment

All animal experiments in the present study conformed to the Guide for the Care and Use of Laboratory Animals published by the United States National Institutes of Health, and were approved by the institutional animal care and use committees of Osaka University Graduate School of Dentistry.

Mouse NAFLD model was produced according to the method described previously [28]. Briefly, C57BL/6J mice (6 weeks old, Charles River Japan, Tokyo) allowed free access to water and food throughout experimental period. Mice were randomly divided three groups, High-fat diet control (HFD control); High-fat diet plus *P. gingivalis *(HFD + P.g); and Basal diet plus *P. gingivalis *(Basal diet + P.g), respectively. HFD groups were administered High-fat diet 32 (Japan CLEA, Tokyo); this feed contains 506.8 kcal/100 g (57.5% from fat, 19.7% from protein, and 22.8% from carbohydrate). Basal diet group was administered basal diet (ORIENTAL YEAST Co., Ltd., Tokyo); this feed contains 360 kcal/100 g (13.3% from fat, 26.2% from protein, and 60.5% from carbohydrate). Four weeks after the start of HF or basal diet, mice were administered *P. gingivalis *(OMZ314 strain, 10^7 ^cells/body) or vehicle via jugular vein. Because of the high-frequency of type II *fimA *detection in NAFLD patients, we selected the OMZ314 strain (Type II *fimA*) for the administration to mice. Twelve weeks after the start of HF or basal diet, body weight, liver weight, and pathological observations were performed.

### Periodontal treatments

We randomly selected 10 NAFLD patients who were also suffering from periodontitis associated with *P. gingivalis*. We prospectively evaluated the efficacy of consecutive periodontal treatments on the amelioration of NAFLD. After provision of adequate explanation, written informed consent was obtained from all the patients prior to their entry into this study. This trial was registered with the University Hospital Medical Information Network (UMIN) Clinical Trial Registry (No. UMIN000004281).

The inclusion criteria for patients with chronic periodontitis were patients who had not received any periodontal therapies within the previous six months or any anti-microbial therapies within the three months prior to the baseline examination, and had at least 10 residual teeth. All of the patients had a PD of greater than 5 mm in at least four tooth regions. Then, oral hygiene instructions were provided to each patient, followed by scaling and root planing procedures, and then application of minocycline hydrochloride. These treatment procedures were continued for 3 months, and the periodontal conditions improved with the treatment. In our previous therapy of periodontal treatments similar to this study, more than 95% of decrease in *P. gingivalis *in oral samples was confirmed.

The patients did not receive any medication for the treatment of impaired liver function, metabolic syndrome or NAFLD during the 3-month periodontal treatment period.

### Statistical analysis

Statistical analyses were performed using the SPSS for Windows, ver. 12. Results are expressed as mean ± SEM. Statistical comparisons were made using the Student's *t*-test or Scheffe's method after analysis of variance (ANOVA). Differences were considered to be significant at < 0.05. In addition, multiple regression analysis was performed in the NAFLD patients and control subjects with *P. gingivalis *infection to identify the predictive value of *P. gingivalis *infection for the development/progression of NFLD, using demographic factors such as the age, history of DM and BMI.

## Results

Frequency of infection with periodontal pathogenic bacteria in NAFLD/NASH patients

**Figure 1:**
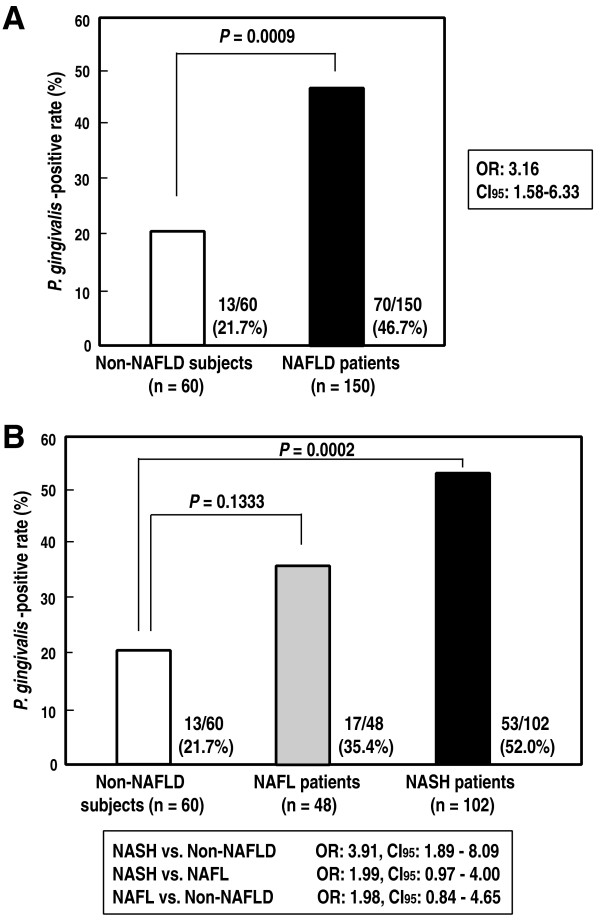
**Detection frequency of *P. gingivalis *in NAFLD/NASH patients and control (non-NAFLD) subjects**. **A: **Comparison of the detection frequency of *P. gingivalis *between NAFLD patients and non-NAFLD (control) subjects. **B: **Comparison of the detection frequencies of *P. gingivalis *among NASH patients, non-alcoholic fatty liver (NAFL) patients and in non-NAFLD (control) subjects. OR: odds ratio, CI: confidence interval.

The detection frequency of *P. gingivalis *infection in the NAFLD patients and control subjects are shown in Table 1. As shown in Figure 1A, the detection frequency of *P. gingivalis *in the NAFLD patients was 46.7%, being significantly higher than that in the non-NAFLD control (healthy) subjects (21.7%; odds ratio: 3.16). Similarly, the detection frequency of *T. denticola *in the NAFLD patients was significantly higher than that in the control subjects (34.7% vs. 16.7%; odds ratio: 2.65). Interestingly, the detection frequency of *P. gingivalis *in the patients with NASH was also markedly higher than that in the non-NAFLD control subjects (52.0%; odds ratio: 3.91, in Figure 1B). In contrast, no significant differences in the detection frequency of any other bacterial species were noted between the NAFLD patients and control subjects (data not shown).

### Multiple regression analysis to identify predictive factors for the development/progression of NAFLD

To clarify the relationship between NAFLD and *P. gingivalis *infection, multiple regression analysis was performed for the NAFLD patients and the control subjects using demographic factors such as the age, history of DM and BMI. The result revealed a statistically significantly higher prevalence of *P. gingivalis *infection in NAFLD patients as compared with that in the control subjects, even after adjusting for age, history of DM and BMI (Table 2). This result suggests that the presence of *P. gingivalis *infection might be an independent predictor of the development of NAFLD.

**Table 2:** Multiple regression analysis to identify predictive factors for the development/progression of NAFLD

Risk Factors	standard error	Odds ratio	*P *value	95% CI
*P. gingivalis*	0.490	2.615	0.049	1.001-6.832
Age	0.014	0.994	0.668	0.968-1.021
DM	0.821	9.433	0.006	1.883-47.62
BMI	0.085	1.631	< 0.0001	1.381-1.927

The dependent variable is presence of NAFLD, and the independent variables are presence/absence of *P. gingivalis *infection, age, presence/absence of diabetes (DM), and body mass index (BMI). *P *value < 0.05 were considered significant. R2 for entire model = 0.429.

### Comparison of various parameters between P. gingivalis-positive and P. gingivalis-negative NAFLD patients

As shown in Table 3, comparative analysis of various parameters was conducted between *P. gingivalis*-positive and *P. gingivalis*-negative NAFLD patients. No significant differences in the serum ALT, AST, γGTP, TG, HDL, LDL, Fe, FPG, IRI, HA or type-IV collagen 7S were observed between the two groups of patients. However, a tendency towards decrease of the serum levels of γGTP, TG, cholinesterase, Fe and ferritin was observed in the *P. gingivalis*-positive patients. In addition, significant decrease of the serum albumin was also observed in this group. Similar results were observed in the *P. gingivalis*-positive and -negative patients with NASH. On the other hand, a significant difference in the prevalence of DM was observed between the *P. gingivalis*-positive and -negative groups among patients with NASH, while no such difference was observed between the two groups among NAFLD patients.

**Table 3:** Comparison of various parameters between *P.gingivalis *(+) and (-) NASH and NAFL patients

		NASH (n = 102)			NAFL (n = 48)	
		
	*P.gingivalis *	*P.gingivalis *	*P *values	*P.gingivalis *	*P.gingivalis *	*P *values
	(+)	(-)		(+)	(-)	
	(n = 53)	(n = 49)		(n = 17)	(n = 31)	
AST (IU/L)	54.8 ± 4.9	54.8 ± 3.7	0.9994	32.1 ± 2.8	39.1 ± 3.1	0.1410
ALT (IU/L)	72.9 ± 6.9	78.9 ± 5.9	0.5179	47.6 ± 6.3	63.8 ± 7.3	0.1472
γGTP (IU/L)	74.0 ± 8.2	71.8 ± 7.8	0.8479	62.1 ± 9.9	116.3 ± 22.7	0.0930
ChoE (IU/L)	357.9 ± 12.0	383.9 ± 13.5	0.1537	397.3 ± 14.7	377.3 ± 12.6	0.3267
Albumin (g/dl)	4.35 ± 0.05	4.50 ± 0.05	0.0488*	4.25 ± 0.09	4.58 ± 0.06	0.0017*
TG (mg/dl)	161.3 ± 11.8	162.4 ± 9.4	0.9246	163.6 ± 21.7	161.5 ± 12.1	0.9293
HDL (mg/dl)	60.2 ± 3.1	54.8 ± 3.1	0.2190	57.0 ± 3.3	58.7 ± 3.4	0.7470
LDL (mg/dl)	122.9 ± 4.0	124.3 ± 5.2	0.8295	132.2 ± 7.9	129.5 ± 5.2	0.7720
Ferritin (ng/ml)	203.8 ± 22.4	276.4 ± 32.4	0.0646	178.6 ± 30.9	211.1 ± 32.2	0.5203
Fe (ng/ml)	110.2 ± 6.5	130.7 ± 11.5	0.1241	107.2 ± 9.6	117.0 ± 8.1	0.4636
FPG (mg/dl)	133.6 ± 25.4	121.9 ± 9.6	0.6483	112.5 ± 7.0	133.7 ± 17.5	0.4282
IRI (μU/ml)	15.1 ± 1.8	15.6 ± 1.6	0.8397	9.5 ± 1.5	11.0 ± 2.1	0.7461
DM (Y:N)	17:36	7:42	0.0386*	5:12	6:25	0.4856
HA (ng/ml)	68.9 ± 11.1	52.3 ± 7.2	0.2178	43.3 ± 15.1	34.2 ± 5.9	0.5046
IV collagen 7S (mg/ml)	5.32 ± 0.22	5.00 ± 0.18	0.2632	3.62 ± 0.17	3.84 ± 0.17	0.2454

DM: with (Y) or without (N) diabetes mellitus

### Analysis of P. gingivalis fimbriae

**Figure 2:**
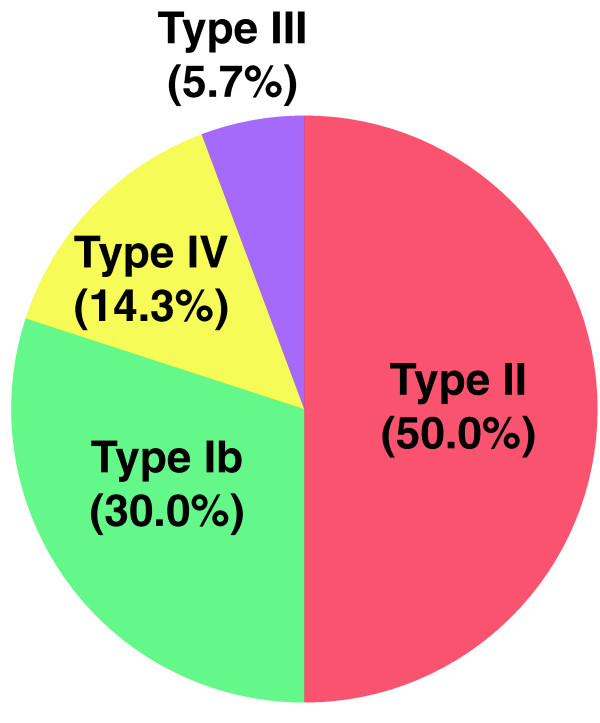
**Percentage of detection frequency of various *fimA *types on NAFLD patients**. Most of the *fimA *genotypes detected in the *P. gingivalis*-positive specimens were of the invasive types; II (50.0%), IV (14.3%), Ib (30.0%); total, 94.3%. Type III (5.7%) is non-invasive type.

*P. gingivalis *fimbriae, filamentous appendages on the bacterial surface, are classified into 6 genotypes based on the diversity of the *fim*A genes encoding each fimbrial subunit. It has been demonstrated that bacterial clones with types II, IV or Ib *fim*A are invasive, whereas those with types I, III or V fimbriae are non-invasive [23,25,26]. Interestingly, most of the *fimA *genotypes detected in the *P. gingivalis*-positive specimens were of the invasive types; II (50.0%), IV (14.3%), Ib (30.0%); total, 94.3% (Figure 2). These results suggest that invasive-type *P. gingivalis *may be involved in the progression of NAFLD.

### Acceleration of NAFLD in HFD-mice infected with P. gingivalis: Analysis of mouse NAFLD model

**Figure 3:**
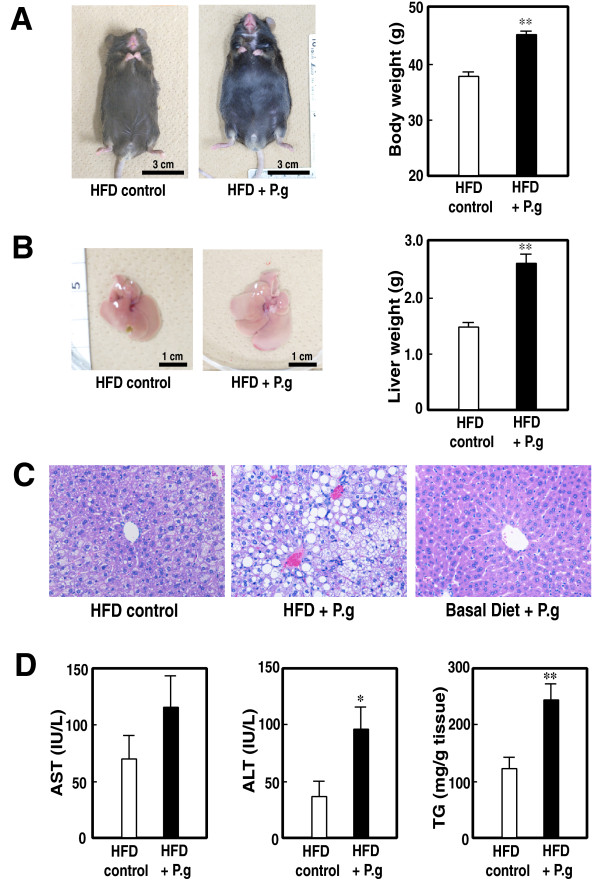
**Effect of administration of *P. gingivalis *on mouse NAFLD model**. **A: **Typical pictures of whole body (left panel) and body weight (right panel) of mice administered vehicle (HFD control) or *P. gingivalis *(HFD + P.g) under the high fat diet (HFD) condition. Each column represents mean + SEM from 12 to 13 independent animals. **; *P *< 0.01. **B: **Typical pictures of liver (left panel) and liver weight (right panel) of mice administered vehicle (HFD control) or *P. gingivalis *(HFD + P.g) under the high fat diet (HFD) condition. Each column represents mean + SEM from 12 to 13 independent animals. **; *P *< 0.01. **C: **Typical pathological pictures of liver of mice administered vehicle (HFD control) or *P. gingivalis *(HFD + P.g) under the high fat diet (HFD) condition, or *P. gingivalis *under basal diet condition (Basal diet + P.g). Each column represents mean + SEM from 12 to 13 independent animals. **; *P *< 0.01. **D: **Alterations of ALT/AST levels and liver triglyceride level. Each column represents mean + SEM from 6 to 9 independent animals. *; *P *< 0.05 and **; *P *< 0.01, respectively.

In our HFD-induced mouse NAFLD model, accumulation of lipid in liver and marked increase in body weight are usually observed at 24 weeks, but slight at 12 weeks, after the start of HFD [28]. However, in this study, we compared body weight, liver weight, and pathological findings between *P. gingivalis*- and vehicle-administered group at 12 weeks after the start of HFD or basal diet. As shown in Figure 3, the mice of HFD control group showed the slight increase in body and liver weight (Figure 3A and 3B, right panels). In contrast, the mice of HFD + P.g group showed the marked increase in body and liver weight. Obvious differences of whole body and liver between HFD control and HFD + P.g group were observed (Figure 3A and 3B, left panels). In contrast, administration of *Streptococcus mutans*, major caries pathogen but non-periodontitis-related oral bacteria, did not show any increases in body (33.50 + 2.51 g on bacteria vs. 36.33 + 3.21 g on control, *P *= 0.9325) and liver weight (1.27 + 0.15 g on bacteria vs. 1.38 + 0.13 g on control, *P *= 0.3811). In addition, other non-virulent oral bacteria, such as *Streptococcus sanguinis *and *Streptococcus salivalius*, did not show the increases in body and liver weights (data not shown). These results indicate that the effect of marked increase in body and liver weight might be *P. gingivalis*-specific phenomena. On the pathological observations, marked accumulation of lipid was observed in the mice infected with *P. gingivalis *(Figure 3C, middle panel). Furthermore, the marked increases in ALT level and liver triglyceride level were also observed (Figure 3D). These results indicate that the infection of *P. gingivalis *on HFD condition accelerates the progression of NAFLD.

### Improvement of liver functions by the periodontal treatments in NAFLD patients with periodontitis

**Figure 4:**
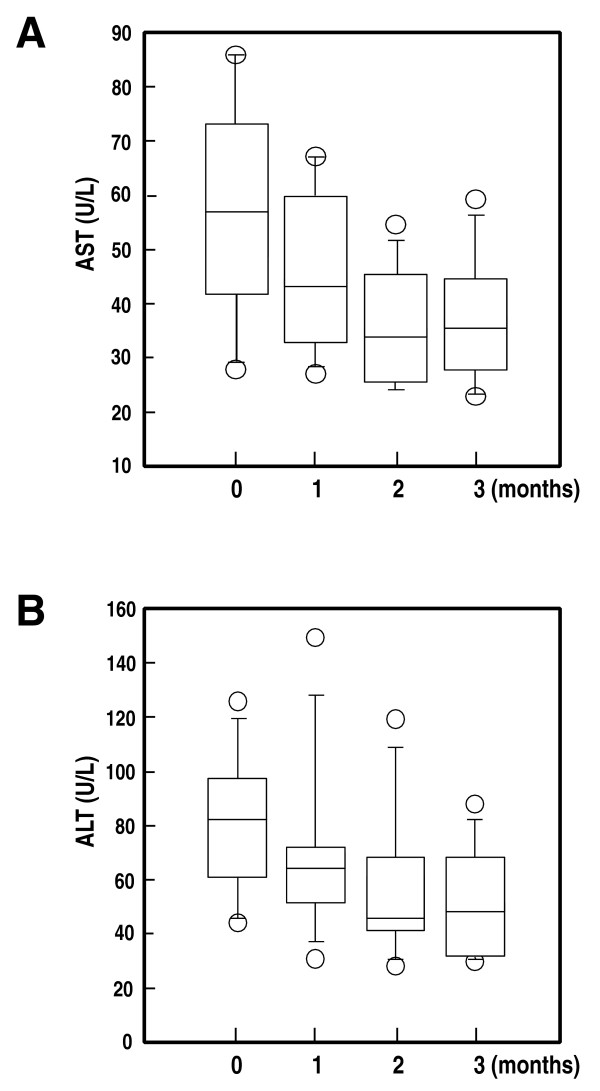
**Improvement of the serum AST and ALT levels with periodontal treatments in NAFLD patients with periodontitis**. Box plots represent the inter-quartile range (boxes), median (central horizontal lines), range (thin lines) and outliers (circles) of the serum AST or ALT levels from 10 cases. Stepwise decreases of the serum AST (*P *< 0.0001, Kruskal-Wallis test) and ALT (*P *< 0.0001, Kruskal-Wallis test) were observed during the course of the periodontal treatments.

To clarify the possibility that periodontal treatments in NAFLD patients with periodontitis may improve the condition of NAFLD, we applied the periodontal treatments to randomly selected NAFLD patients. A tendency towards decrease of the serum AST and ALT levels with periodontal treatments was observed by 1 month after the start of the treatment procedures (Figure 4). At the period of 2 months of treatments, significant decreases of both serum AST and ALT levels were observed as compared with the levels at the baseline (*P *= 0.0165, and *P *= 0.0031, respectively). Further improvements of the serum AST and ALT levels were observed at the end of 3 months after the start of the treatment procedures (*P *= 0.0069, and *P *= 0.0009, respectively). There were no changes in body weights during the treatment period.

## Discussion

Many factors have been implicated in the pathogenesis of NAFLD/NASH, including obesity, insulin resistance, oxidative stress and mitochondrial dysfunction [1-6]. Recently, the association between low vitamin D level and NAFLD has also been reported [29]. However, it is still unclear whether any other factors might be involved in the pathogenesis and progression of NASH in its common form. Therefore, identifying the mechanisms responsible for the progression of NASH may be useful for designing therapeutic strategies for these diseases.

In the present study, we showed that the prevalence of *P. gingivalis *infection was significantly higher in the NAFLD patients than in the healthy subjects. This result suggests that *P. gingivalis *infection may be involved in the mechanism of onset of NAFLD, because *P. gingivalis *itself or the endotoxin and cytokines released from the bacteria can easily enter the blood circulation. Multiple regression analysis in NAFLD patients and control subjects to identify the predictive value of *P. gingivalis *infection for the development of NAFLD using demographic factors such as the age, history of DM and BMI revealed a significantly higher prevalence of *P. gingivalis *infection in NAFLD patients as compared with that in control subjects, even after adjusting for age, history of DM and BMI. This result suggests that *P. gingivalis *infection may be an independent risk factor for NAFLD.

A relationship has been reported between infection with periodontal bacteria and the onset of type 2 diabetes mellitus [30,31]. Namely, it is considered that the increased serum levels of lipopolysaccharide and TNF-α associated with *P. gingivalis *infection induce insulin resistance, leading to the development of type 2 diabetes [30,31]. In addition, our colleagues reported relationship between the fimbrial type of the periodontal bacteria causing periodontitis and the risk of development of type 2 diabetes mellitus [32]. In fact, most NAFLD patients with *P. gingivalis *infection show bacteria with invasive types of fimbria, such as II, IV, and Ib. Therefore, it is suggested that the high detection frequency of *P. gingivalis *in NAFLD patients may be due to the presence of coexisting DM, based on the correlation between NAFLD and DM. However, in the present study, no significant difference in the frequency of DM was noted between *P. gingivalis*-positive and -negative groups among the NAFLD patients (Table 3). Therefore, it is suggested that the high detection frequency of *P. gingivalis *in NAFLD patients was not due to coexisting DM in our study. In contrast, a statistically significant difference in the frequency of DM was noted between the *P. gingivalis*-positive and -negative NASH patients. These results may indicate that both DM and *P. gingivalis *infection may be involved in the progression of NAFL to NASH. Namely, both DM and *P. gingivalis *infection may cooperatively increase the risk of progression from NAFL to NASH. In fact, the high detection frequency of *P. gingivalis *infection in NASH patients was more obvious than that in the NAFL patients.

In comparison between *P. gingivalis*-positive and negative NASH/NAFLD patients, statistically significant decrease in serum albumin level was observed. These results indicate that decrease in liver function may be accelerated in *P. gingivalis*-positive patients. In addition, the tendency, but not significant, of increases in hyaluronic acid and IV collagen 7S levels were also observed. As those are indices of the progression of liver fibrosis, it may be hypothesized that liver fibrosis and decrease in function might be accelerated in *P. gingivalis*-positive patients, although the destroy of liver is not so marked.

Thus, infection with *P. gingivalis *may be one of the risk factors for not only the second stage of progression to NASH, but also the first stage of the pathogenesis for NAFL. In fact, the infection of type II *P. gingivalis *on NAFLD mouse model dramatically accelerated the NAFLD progression without any other additional treatments such as choline-deficient, l-amino acid-defined diet-fed [33] or LDL receptor knockout [34]. The NAFLD progression on *P. gingivalis*-infected mice was markedly faster than that on control mice under the HFD condition, but not basal diet condition (see Figure 3C, right panel). These results clearly indicate that both HFD condition and *P. gingivalis *infection cooperatively increase the risk of pathogenesis of NAFLD. As the infection of other oral bacteria on NAFLD model did not accelerate the progression to NAFLD, the acceleration of NAFLD progression by *P. gingivalis *under HFD condition might be high-virulence *P. gingivalis*-specific effect. Further animal experiments will be required.

The mechanism of *P. gingivalis*-mediated the pathogenesis of NAFLD/NASH is unclear. The current model of NASH pathogenesis proposes two stages of progression. First, insulin resistance causes lipid accumulation in the hepatocytes; second, cellular insults, such as oxidative stress, lipid toxicity, mitochondrial dysfunction, and/or bacterial endotoxins from the gut cause hepatic inflammation, resulting in the development of NASH [1-6]. In fact, administration of lipopolysaccharide (LPS) showed the NASH-like conditions in HFD mice (unpublished data). Because the infection with high-virulence strains of *P. gingivalis *may generate a large amount of lipopolysaccharide and TNF-α, it may result in inflammation of not only the local gingiva, but also involve other systemic organs [23,25,30,31]. Such inflammatory mediators are involved in insulin resistance. In addition, *P. gingivalis *can easily invade the blood circulation from the gingiva after several periodontal procedures/processes, including tooth brushing, chewing, subgingival irrigation, and dental extractions [9-11]. These reports support our conclusions that infection with *P. gingivalis *may be one of the risk factors for the development of NAFLD/NASH.

We also confirmed the efficacy of the periodontal treatments in improving liver function parameters such as serum AST and ALT in NAFLD patients (Figure 4). This result indicates that periodontitis caused by *P. gingivalis *in NAFLD patients may be a risk factor for the aggravation of NAFLD, and that periodontal treatments may be useful supportive measures in the management of patients with NAFLD. Further large scale clinical practice for the periodontal treatments in NAFLD patients will be required in future.

## Conclusions

In conclusion, *P. gingivalis *infection was noted at a significantly high frequency in NAFLD/NASH patients. Infection of type II *P. gingivalis *on NAFLD mouse model accelerated the NAFLD progression. Also, the effectiveness of periodontal treatments in ameliorating the severity of NAFLD was observed. Thus, infection with high-virulence *P. gingivalis *may be a risk factor for the development/progression of NAFLD/NASH.

## Abbreviations

NASH: Nonalcoholic steatohepatitis; NAFLD: Nonalcoholic fatty liver disease; *P. gingivalis*: *Porphyromonas gingivalis*; AST: Aspartate aminotransferase; ALT: Alanine aminotransaminase; GGT: Gamma glutamyltranspeptidasehigh; HDL: High density lipoprotein; LDL: Low density lipoprotein; PCR: Polymerase chain reaction.

## Competing interests

The authors declare that they have no competing interests.

## Authors' contributions

MY, KN and KW were involved in the conception and design of this study under the supervision of TO, AA, TO and AN. Clinical sample collections and suggestions for NAFLD patients were provided by HE, HM, KI, MO, YS and YS. Sample analysis and evaluation were performed by SN, RN, KH and KU. Animal experiment was performed by SN, KW, KH, MK and KU. Periodontal treatments were performed by MY, SM and IT. YM, KN, SN and RN performed the statistical analyses and interpretation of the results under the supervision of KW. YM, KN and KW wrote the manuscript. All authors read the manuscript and approved for submission.

## Pre-publication history

The pre-publication history for this paper can be accessed here:

http://www.biomedcentral.com/1471-230X/12/16/prepub

## References

[B1] Angulo P Nonalcoholic fatty liver disease N Engl J Med 2002 18 1221 1231 10.1056/NEJMra011775 11961152

[B2] Ludwig J Viggiano TR McGill DB Oh BJ Nonalcoholic steatohepatitis: Mayo Clinic experiences with a hitherto unnamed disease Mayo Clin Proc 1980 55 434 438 7382552

[B3] Liou I Kowdley KV Natural history of nonalcoholic steatohepatitis J Clin Gastroenterol 2006 40 (Suppl 1):S11 S16 10.1097/01.mcg.0000168644.23697.31 16540761

[B4] Diehl AM Goodman Z Ishak KG Alcohol-like liver disease in nonalcoholics. A clinical and histologic comparison with alcohol-induced liver injury Gastroenterology 1998 95 1056 1062 3410220

[B5] Abdelmalek MF Diehl AM Nonalcoholic fatty liver disease as a complication of insulin resistance Med Clin North Am 2007 91 1125 1149 10.1016/j.mcna.2007.06.001 17964913

[B6] Marchesini G Bugianesi E Forlani G Cerrelli F Lenzi M Manini R Nonalcoholic fatty liver, steatohepatitis, and the metabolic syndrome Hepatology 2003 37 917 923 10.1053/jhep.2003.50161 12668987

[B7] Tonetti MS D'Aiuto F Nibali L Donald A Storry C Parkar M Treatment of periodontitis and endothelial function N Engl J Med 2007 356 911 920 10.1056/NEJMoa063186 17329698

[B8] D'Aiuto F Parkar M Andreaou G Brett PM Ready D Tonetti MS Periodontitis and atherogenesis: causal association or simple coincidence? J Clin Periodontol 2004 31 402 411 10.1111/j.1600-051X.2004.00580.x 15086624

[B9] Sconyer JR Crawford JJ Moriarty JD Relationship of bacteremia to tooth brushing in patients with periodontitis J Am Dent Assoc 1973 87 616 622 4516507 10.14219/jada.archive.1973.0453

[B10] Caroll GC Sebor RJ Dental flossing and its relationship to transient bacteremia J Periodontol 1980 51 691 692 6937641 10.1902/jop.1980.51.12.691

[B11] Forner L Larsen T Kilian M Holmstrup P Incidence of bacteremia after chewing, tooth brushing and scaling in individuals with periodontal inflammation J Clin Periodontol 2006 33 401 407 10.1111/j.1600-051X.2006.00924.x 16677328

[B12] Scannapieco FA Bush RB Paju S Associations between periodontal disease and risk for atherosclesosis, cardiovascular disease, and stroke. A systematic review Ann Periodontol 2003 8 38 53 10.1902/annals.2003.8.1.38 14971247

[B13] Beck J Garcia R Heiss G Vokonas PS Offenbacher S Periodontal disease and cardiovascular disease J Periodontol 1996 67 1123 1137 8910831 10.1902/jop.1996.67.10s.1123

[B14] Offenbacher S Madianos PN Champagne CM Southerland JH Paquette DW Williams RC Periodontitis-atherosclerosis syndrome: an expanded model of pathogenesis J Periodontal Res 1999 34 346 352 10.1111/j.1600-0765.1999.tb02264.x 10685359

[B15] Matteoni CA Younossi ZM Gramlich T Boparai N Liu YC McCullough AJ Nonalcoholic fatty liver disease: a spectrum of clinical and pathological severity Gastroenterology 1999 116 1413 1419 10.1016/S0016-5085(99)70506-8 10348825

[B16] Brunt EM Nonalcoholic steatohepatitis: definition and pathology Semin Liver Dis 2001 21 3 16 10.1055/s-2001-12925 11296695

[B17] Brunt EM Janney CG Di Bisceglie AM Neuschwander-Tetri BA Bacon BR Nonalcoholic steatohepatitis: a proposal for grading and staging the histological lesions Am J Gastroenterol 1999 94 2467 2474 10.1111/j.1572-0241.1999.01377.x 10484010

[B18] Fujita K Nozaki Y Wada K Yoneda M Fujimoto Y Fujitake M Dysfunctional very-low-density lipoprotein synthesis and release is a key factor in nonalcoholic steatohepatitis pathogenesis Hepatology 2009 50 722 780 10.1002/hep.23094 19650159

[B19] Mofrad P Contos MJ Haque M Sargeant C Fisher RA Luketic VA Clinical and histologic spectrum of nonalcoholic fatty liver disease associated with normal ALT Hepatology 2003 37 1286 1292 10.1053/jhep.2003.50229 12774006

[B20] Lee JY Kim KM Lee SG Yu E Lim YS Lee HC Prevalence and risk factors of non-alcoholic fatty liver disease in potential living liver donors in Korea: a review of 589 consecutive liver biopsies in a single center J Hepatol 2007 47 239 244 10.1016/j.jhep.2007.02.007 17400323

[B21] Fracanzani AL Valenti L Bugianesi E Andreoletti M Colli A Vanni E Risk of severe liver disease in nonalcoholic fatty liver disease with normal aminotransferase levels: a role for insulin resistance and diabetes Hepatology 2008 48 792 798 18752331 10.1002/hep.22429

[B22] Matthews DR Hosker JP Rudenski AS Homeostasis model assessment: insulin resistance and beta-cell function from fasting plasma glucose and insulin concentrations in man Diabetologia 1985 28 412 419 10.1007/BF00280883 3899825

[B23] Amano A Nakagawa I Kataoka K Morisaki I Hamada S Distribution of *Porphyromonas gingivalis *strains with *fimA *genotypes in periodontitis patients J Clin Microbiol 1999 37 1426 1430 10203499 10.1128/jcm.37.5.1426-1430.1999 PMC84792

[B24] Tran SD Rudney JD Multiplex PCR using conserved and species-specific 16S rRNA gene primers for simultaneous detection of *Actinobacillus actinomycetemcomitans *and *Porphyromonas gingivalis* J Clin Microbiol 1996 34 2674 2678 8897163 10.1128/jcm.34.11.2674-2678.1996 PMC229384

[B25] Nakagawa I Amano A Ohara-Nemoto Y Endoh N Morisaki I Kimura S Identification of a new variant of *fimA *gene of *Porphyromonas gingivalis *and its distribution in adults and disabled populations with periodontitis J Periodontal Res 2002 37 425 432 10.1034/j.1600-0765.2002.01637.x 12472836

[B26] Nakagawa I Amano A Kimura RK Nakamura T Kawabata S Hamada S Disribution and molecular characterization of *Porphyromonas gingivalis *carrying a new type of *fimA *gene J Clin Microbiol 2000 38 1909 1914 10790120 10.1128/jcm.38.5.1909-1914.2000 PMC86621

[B27] Nakano K Inaba H Nomura R Nemoto H Takeda M Yoshioka H Detection of cariogenic *Streptococcus mutans *in extirpated heart valve and atheromatous plaque specimens J Clin Microbiol 2006 44 3313 3317 10.1128/JCM.00377-06 16954266 PMC1594668

[B28] Nozaki Y Fujita K Yoneda M Wada K Shinohara Y Takahashi H Long-term combination therapy of ezetimibe and acarbose for non-alcoholic fatty liver disease J Hepatol 2009 51 548 556 10.1016/j.jhep.2009.05.017 19596472

[B29] Barchetta I Angelico F Ben MD Baroni MG Pozzilli P Morini S Strong association between non alcoholic fatty liver disease (NAFLD) and low 25(OH) vitamin D levels in an adult population with normal serum liver enzymes BMC Med 2011 9 85 10.1186/1741-7015-9-85 21749681 PMC3148980

[B30] Loe H Periodontal disease: the sixth complication of diabetes mellitus Diabetes Care 1993 16 329 334 8422804

[B31] Nelson RG Shlossman M Budding L Pettitt DJ Saad MF Genco RJ Periodontal disease and NIDDM in Pima Indians Diabetes Care 1990 13 836 840 10.2337/diacare.13.8.836 2209317

[B32] Ojima M Takeda M Yoshioka H Nomura M Tanaka N Kato T Relationship of periodontal bacterium genotypic variations with periodontitis in type 2 diabetic patients Diabetes Care 2005 28 433 434 10.2337/diacare.28.2.433 15677809

[B33] Fujita K Nozaki Y Yoneda M Wada K Takahashi H Kirikoshi H Nitric oxide plays a crucial role in the development/progression of nonalcoholic steatohepatitis in the choline-deficient, l-amino acid-defined diet-fed rat model Alcohol Clin Exp Res 2010 34 Suppl 1 S18 24 18986378 10.1111/j.1530-0277.2008.00756.x

[B34] Hoekstra M Li Z Kruijt JK Van Eck M Van Berkel TJC Kuiper J The expression level of non-alcoholic fatty liver disease-related gene PNPLA3 in hepatocytes is highly influenced by hepatic lipid status J Hepatol 2010 52 244 251 10.1016/j.jhep.2009.11.004 20015565

